# Lifestyle factors and clustered patterns of body shape change in Korean adults: A longitudinal observational study

**DOI:** 10.1371/journal.pone.0360853

**Published:** 2026-10-15

**Authors:** Jaeho Lee, Kyoungsik Jeong, Youngseop Lee, Younghwa Baek

**Affiliations:** Division of Korean Medicine Data, Korea Institute of Oriental Medicine, Daejeon, Republic of Korea; Nanjing Medical University Affiliated Nanjing Municipal Center for Disease Control and Prevention, CHINA

## Abstract

**Background:**

Obesity is a major health problem driven by lifestyle habits and changes in body composition, particularly increased body fat mass and reduced skeletal muscle mass. Body mass index alone cannot fully characterize adiposity distribution or changes in muscle mass. Body-shape indicators such as waist circumference, waist-to-hip ratio, and waist-to-height ratio predict metabolic and cardiovascular disease risk; however, few studies have assessed longitudinal changes in these measures together with lifestyle factors. In this study, we aimed to identify clusters of body-shape change using 2-year follow-up data from Korean adults and to elucidate the body-composition and lifestyle factors underlying these changes.

**Methods:**

K-means cluster analysis was applied to 1,404 participants in the Korean Medicine Daejeon Citizen Cohort using 2-year changes in body mass index, waist circumference, hip circumference, waist-to-hip ratio, waist-to-height ratio, and body weight. Baseline body shape, body composition, and lifestyle indicators were compared across clusters, and multinomial logistic regression was used to identify variables associated with cluster membership.

**Results:**

Three clusters were identified: Stable (48.0%), Adiposity-Increasing (31.0%), and Adiposity-Decreasing (21.0%). At baseline, cluster membership was significantly associated with body fat mass. Over follow-up, changes in body fat mass and skeletal muscle mass showed strong associations with cluster assignment. In addition, clinically meaningful worsening in sleep quality (increase of ≥2.5 points in the Pittsburgh Sleep Quality Index) and reduced physical activity (decrease in activity as measured by the Global Physical Activity Questionnaire) were significantly associated with the Adiposity-Increasing cluster.

**Conclusions:**

Changes in body composition, especially body fat mass and skeletal muscle mass, were closely associated with body-shape trajectories, while worsening sleep quality and declining physical activity were associated with the Adiposity-Increasing trajectory. These findings support an integrative approach that considers body shape, body composition, and modifiable lifestyle factors when characterizing adiposity-related trajectories and designing tailored lifestyle interventions.

## Introduction

Overweight and obesity negatively affect nearly all physiological functions through the accumulation of abnormal or excessive adipose tissue, leading to increased health risks. Nearly one-third of the global population is currently classified as either overweight or obese [1].

Obesity results from complex interactions among multiple factors, including genetics, body composition and fat distribution, lifestyle habits, and psychological factors. Rather than acting independently, these factors influence one another and may increase or decrease obesity risk [2,3]. In particular, lifestyle factors such as sleep and physical activity are closely associated with body mass index (BMI) and may affect obesity risk [3,4]. However, BMI alone is insufficient to capture the health effects of excess adiposity, and additional measures should be considered [5]. Individuals with the same BMI can have different risks of cardiovascular disease, metabolic syndrome, or type 2 diabetes depending on body fat distribution and/or differences in lean mass (including changes in muscle mass) [6,7]. Accordingly, beyond absolute body-weight measures, tracking changes in body shape has been proposed as a more realistic approach with improved predictive power [8–10].

Changes in body shape may reflect not only physiological changes but also changes in lifestyle habits [11]. Decreased sleep quality and physical activity, along with increased sedentary time, are associated with increased fat mass and decreased muscle mass, which may in turn affect body-shape indicators [12,13]. Even individuals with similar body-shape indicators can experience different health effects depending on interactions with lifestyle factors [14]. For example, when increased body weight is accompanied by regular resistance exercise, muscle mass is more likely to increase; however, when accompanied by poor sleep quality or prolonged sedentary behavior, the same weight gain may be more consistent with increased fat mass [13–17].

Given these complex interactions, there is a need to simultaneously analyze changes in body shape and lifestyle factors. Compared with BMI, metrics such as waist-to-height ratio (WHtR), waist-to-hip ratio (WHR), and waist circumference (WC) have been reported to better predict abdominal obesity and metabolic risk [18–20]. BMI alone has also been shown to be insufficient to fully explain adiposity-related risk, and the use of BMI in parallel with measures such as WC and WHtR has been recommended [21]. Thus, combinations of body-shape indicators or patterns of change may improve predictive power for obesity risk and related diseases [21–23].

Nevertheless, most studies to date have evaluated obesity using cross-sectional body-shape indicators, and relatively few analyses account for changes in body shape over time and their interactions with lifestyle factors [24]. Moreover, prior studies have largely focused on linear relationships between a single variable and obesity-related risk, which may not fully reflect the complexity and heterogeneity of health states [21].

Therefore, this study aimed to derive clusters based on changes in body shape over a 2-year follow-up period and to analyze their relationships with underlying body-shape indicators and lifestyle factors, with the goal of providing foundational data for predicting obesity risk and informing customized intervention strategies.

## Materials and methods

### Participants

This study used data from the Korean Medicine Daejeon Citizen Cohort (KDCC), which is managed by the Korean Medicine Data Center (KDC) of the Korea Institute of Oriental Medicine (KIOM) [25]. The KDCC is a prospective cohort study designed to support the prevention and management of chronic diseases and incorporates individual and lifestyle characteristics. The baseline survey was conducted between 2017 and 2019, and follow-up surveys are conducted at 2-year intervals [25]. The first follow-up examinations (visit 2, V2) were conducted between June 2020 and March 2022, during a period that overlapped with the coronavirus disease 2019 (COVID-19) pandemic in Korea. Follow-up assessments were performed at the cohort examination center under standardized protocols in accordance with national COVID-19–related public health and infection-control guidelines. Of the 2,000 participants enrolled at baseline (visit 1, V1), 1,404 participants who completed the first follow-up survey (visit 2, V2; June 2020 to March 2022) and had no missing values for the main variables (body-shape indicators and lifestyle habits) were included in the final analysis (Fig 1). At baseline, we also compared participants included in the analytic sample (n = 1,404) with those excluded because of loss to follow-up and/or missing data (n = 596; data not shown). There were no significant differences in height, weight, BMI, WC, WHR, WHtR, body fat mass, or SMM between the groups (all *p* > .05). The analytic sample was slightly older and had a slightly larger hip circumference than excluded participants (both *p* < .05). All analyses were therefore conducted using a complete-case dataset; selection bias cannot be ruled out if participants with missing data differed systematically from those included.

**Fig 1:**
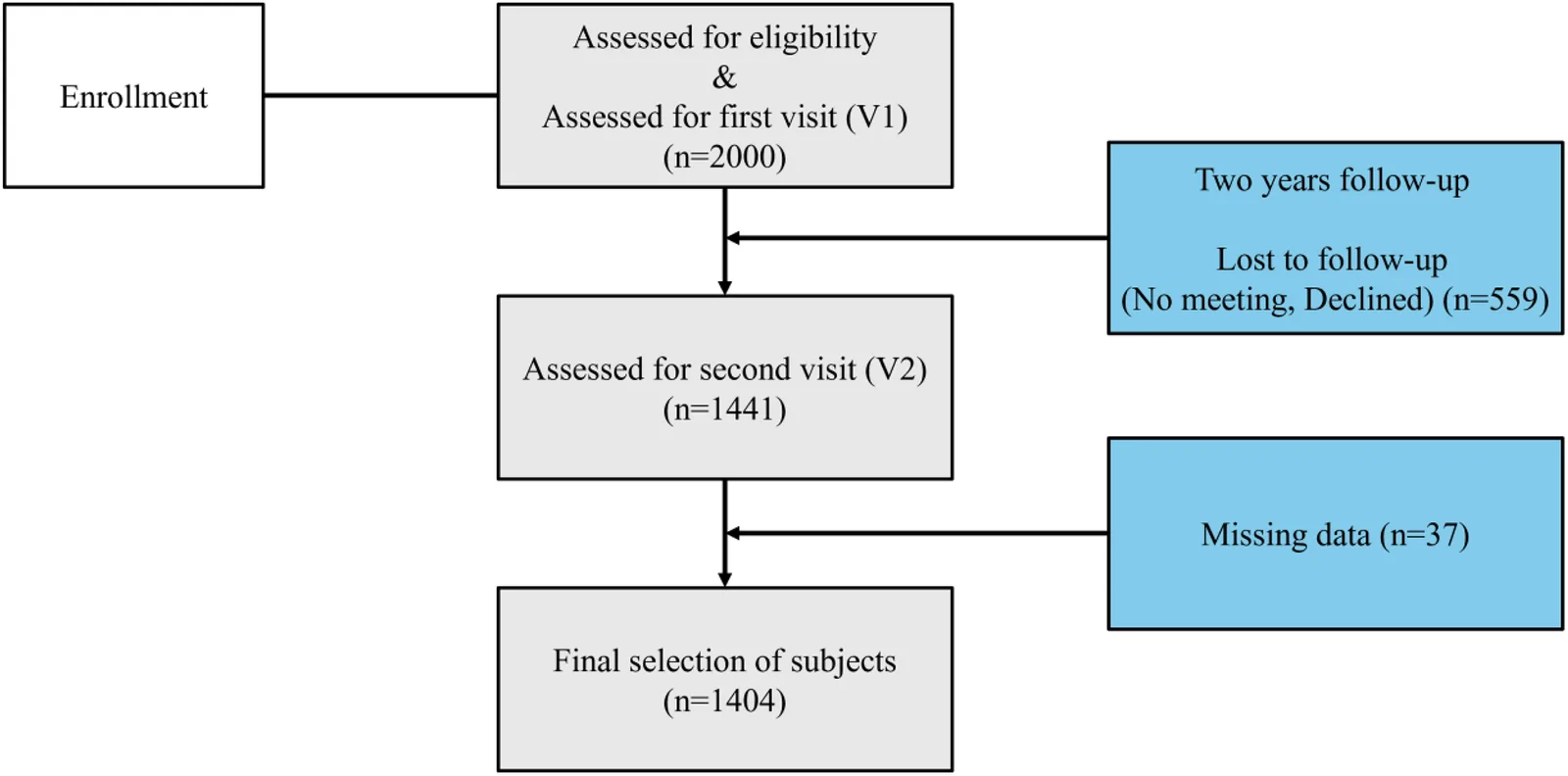
Flowchart of the study.

The mean age of included participants was 44.74 years (SD, 6.67); 421 (30.0%) were male, and 983 (70.0%) were female. All participants provided written informed consent after receiving an explanation of the study objectives and the use of personal information. This study was approved by the KIOM Institutional Review Board (IRB; No. I-1703/002–002, DJDSKH-17-BM-12) and was conducted in accordance with the ethical principles of the Declaration of Helsinki.

### Data collection

Anthropometric measurements, physiological testing, and questionnaire surveys were performed using identical protocols at both V1 and V2 by trained examiners using the same equipment. Devices were regularly maintained and calibrated in accordance with the manufacturer’s recommendations to ensure consistency across time points. Change (Δ) in each index was calculated as the V2 value minus the V1 value.

1) Body shape indicators

BMI, WC, hip circumference (HC), WHR, and WHtR were included as body-shape indicators. Height (cm) and weight (kg) were measured using the BSM370 (Biospace Co., Ltd., Seoul, Korea). BMI was calculated as weight (kg) divided by height (m) squared (kg/m^2^) and, according to the WHO criteria, was classified as underweight, healthy, overweight, or obese [26]. WC and HC were measured using a tape measure (Rollfix, Hoechstmass Co., Sulzbach, Germany) in accordance with WHO guidelines [25]. WC reflects visceral and subcutaneous abdominal fat and is associated with morbidity and premature mortality [27]. HC reflects gluteofemoral fat and lower-limb musculature and may have a protective role relative to WC [27]. WHtR was calculated as WC (cm) divided by height (cm) and has been reported to predict cardiovascular and metabolic disease across diverse populations [22]. WHR was calculated as WC (cm) divided by HC (cm) and is sensitive to abdominal obesity and metabolic risk [28].

2) Lifestyle indicators

Sleep quality and physical activity level were used as lifestyle indicators. Sleep quality was assessed using the Pittsburgh Sleep Quality Index (PSQI), and physical activity level was assessed using the Global Physical Activity Questionnaire (GPAQ). The PSQI assesses sleep quality and has been reported to correlate with changes in health status and body composition [29]. The GPAQ grades physical activity levels based on WHO criteria and is widely used in epidemiologic studies [30].

3) Body-composition indicators

Skeletal muscle mass (SMM) and body fat mass (BFM) were included as body-composition indicators. Body composition was assessed using the InBody 770 (InBody Co., Ltd., Seoul, Korea) via bioelectrical impedance analysis at six frequencies (1, 5, 50, 250, 500 kHz, and 1 MHz) and at five sites (right arm, left arm, trunk, right leg, and left leg) [25]. SMM represents the absolute mass of skeletal muscle and is associated with chronic disease development, including metabolic syndrome [31]. BFM represents the absolute mass of body fat, is influenced by genetic and environmental factors, and is associated with metabolic abnormalities and cardiovascular risk [32].

### Statistical methods

K-means cluster analysis was performed based on changes in body shape (ΔBMI, ΔWC, ΔHC, ΔWHR, ΔWHtR, and ΔWeight). The number of clusters was set to k = 3 based on the elbow plot and the conventional classification of body-shape change into increase, maintenance, and decrease. In the elbow plot, the sum of squared errors (SSE) was 39,896 for 2 clusters, 30,075 for 3 clusters, 25,228 for 4 clusters, and 23,254 for 5 clusters (Fig 2). Cluster labels were assigned according to the predominant direction of change in these indicators over the 2-year follow-up period (increase, stability, or decrease), rather than baseline adiposity. Accordingly, Cluster 1 was named the Stable group, Cluster 2 the Adiposity-Increasing group, and Cluster 3 the Adiposity-Decreasing group. These six change indicators were entered into the K-means algorithm in their original measurement units without prior standardization; given the scale sensitivity of K-means clustering, this approach is a methodological limitation. As a sensitivity analysis, K-means clustering was repeated using Z-score standardized versions of the same six body-shape change indicators, with k fixed at 3. Agreement between the primary unstandardized solution and the standardized-variable solution was assessed using cross-tabulation. Because the clusters were derived solely from changes in body-shape indicators, baseline body-shape, body-composition, and lifestyle variables (V1 BMI, WC, HC, WHR, WHtR, BFM, SMM, PSQI, and GPAQ), as well as age and sex, were treated as external variables for characterizing and interpreting the resulting groups and as candidate predictors in subsequent regression models.

**Fig 2:**
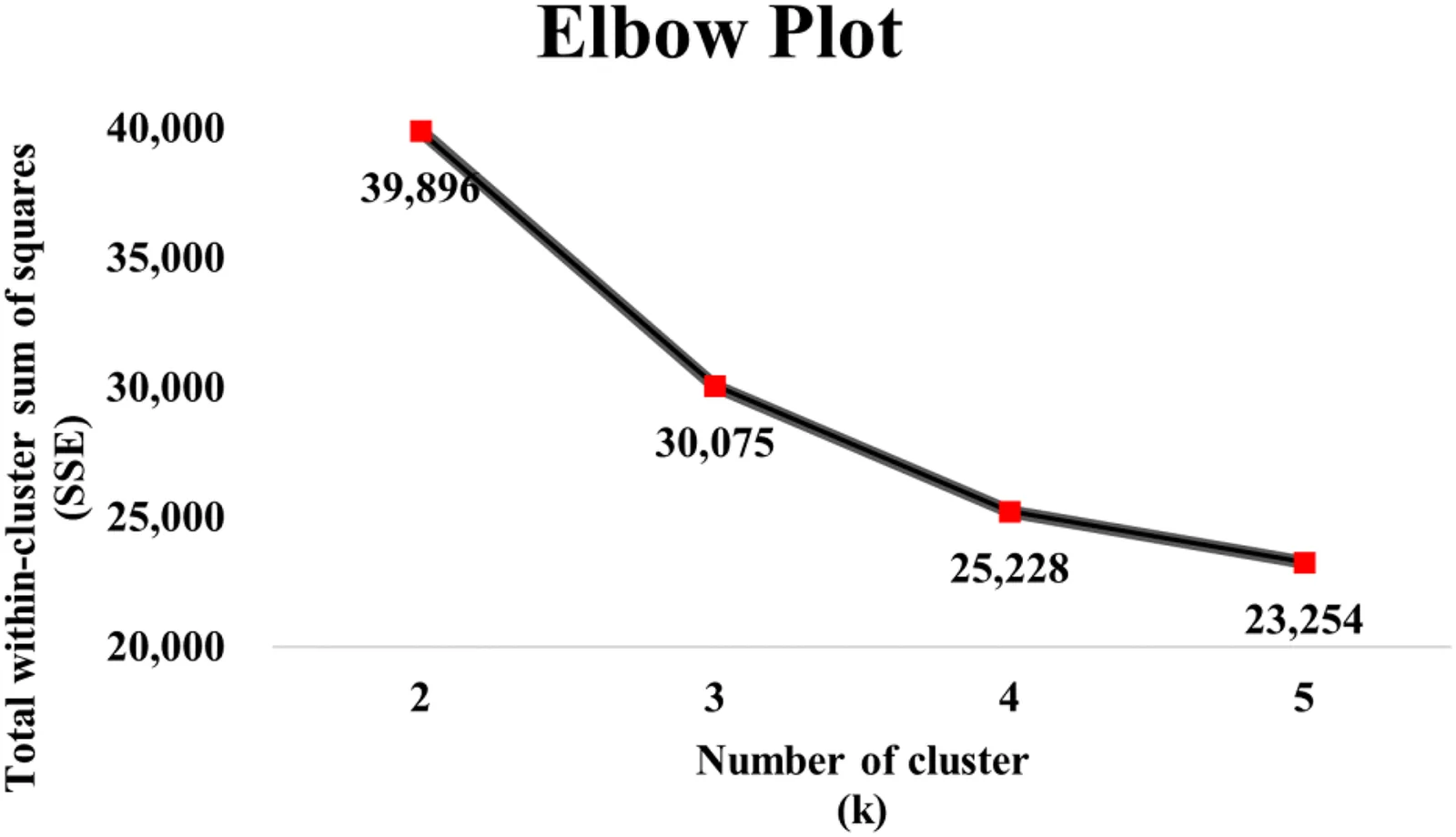
Elbow plot for determining the optimal number of clusters. A distinct elbow is observed at k = 3, where the reduction in SSE begins to level off.

To compare characteristics between clusters, the chi-square (χ^2^) test was used for categorical variables (reported as n [%]), and one-way analysis of variance (ANOVA) was used for continuous variables (reported as mean ± standard deviation). Prior to analysis, normality (Shapiro–Wilk test and Q–Q plots) and homoscedasticity (Levene’s test) were assessed for all continuous variables. Tukey’s honestly significant difference (HSD) test was used for pairwise comparisons when the assumptions of ANOVA were satisfied, and the Games–Howell post-hoc test was used when the equal-variance assumption was violated.

Factors associated with cluster membership were examined using multinomial logistic regression, and odds ratios (ORs) with 95% confidence intervals (CIs) were calculated. Cluster 3 served as the reference group. Associations of both baseline indicators (V1) and changes in indicators with cluster membership were evaluated by including body-shape and lifestyle indicators as independent variables and comparing Clusters 1 and 2 with Cluster 3. Body-shape and lifestyle indicators were included as independent variables. For each analysis, two models were fitted: a crude model and a model adjusted for age and sex. Multicollinearity among the independent variables was assessed using variance inflation factors (VIFs). All VIF values ranged from 1.01 to 4.82 and were below the commonly used threshold of 5.0, indicating that multicollinearity was not a major concern. Model fit was evaluated using the −2 log-likelihood, likelihood-ratio χ^2^ tests, the Hosmer–Lemeshow goodness-of-fit test, and pseudo-R^2^ indices (Nagelkerke R^2^ and McFadden R^2^).

GPAQ scores were standardized (z-scores) prior to analysis, and quartiles were used in some analyses (Table 5). For baseline analyses (Table 4), the PSQI was dichotomized using a cutoff score of 5 (≥5 = poor sleep; < 5 = good sleep) [33]. For analyses of change (Table 5), ΔPSQI ≥ 2.5 was defined as a clinically meaningful change based on the minimal clinically important difference (MCID) [34].

All analyses were performed using IBM SPSS Statistics, version 21.0 (IBM Corp., Armonk, NY, USA). Statistical significance was set at *p* < .05.

## Results

### Cluster analysis based on changes in body shape

K-means clustering (k = 3) was used to categorize the 1,404 participants into three groups. Cluster 1 consisted of 674 participants (48.0%) who showed minimal change in body-shape indicators and was named the Stable group. Cluster 2 consisted of 435 participants (31.0%) who showed an overall increase in body-shape indicators and was named the Adiposity-Increasing group. Cluster 3 consisted of 295 participants (21.0%) who showed an overall decrease in body-shape indicators and was named the Adiposity-Decreasing group.

Centroid values for body-shape change indicators showed that Cluster 2 had the largest increases in key indicators (ΔBMI = 1.21; ΔWC = 7.23), whereas Cluster 3 had the largest decreases (ΔBMI = −1.18; ΔWC = −5.30). Cluster 1 showed minimal change across indicators (Table 1). Thus, cluster names reflect trajectory patterns over time rather than baseline adiposity. Notably, Cluster 3 was labeled Adiposity-Decreasing because it showed the greatest reductions in body-shape indices despite having the highest baseline adiposity. In the sensitivity analysis using Z-score standardized body-shape change indicators, the three-cluster structure was broadly retained, corresponding to the Stable, Adiposity-Increasing, and Adiposity-Decreasing groups (S1 Table). The standardized-variable solution showed high agreement with the primary unstandardized solution, with an overall agreement of 93.0% and cluster-specific agreement ranging from 90.8% to 93.8% (S2 Table).

**Table 1:** Cluster classification based on changes in body shape parameters (n = 1,404).

Variables	Cluster 1 (Stable)	Cluster 2 (Adiposity-Increasing)	Cluster 3 (Adiposity-Decreasing)
Total n (%)	674 (48.0%)	435 (31.0%)	295 (21.0%)
Cluster centroids			
ΔBMI	0.04	1.21	−1.18
ΔWHR	0.01	0.04	−0.03
ΔWHtR	0.01	0.04	−0.03
ΔWC	1.37	7.23	−5.30
ΔHC	0.73	3.92	−2.69
ΔWeight	0.29	3.43	−3.02

Cluster centroids of body shape change variables

### Comparison of baseline indicators between clusters

Baseline (V1) indicators differed significantly across clusters for most variables (Table 2). Sex distribution differed by cluster (χ^2^ test, *p* = .009), with a lower proportion of male participants in Cluster 3 (22.7%) than in Cluster 1 (31.8%) and Cluster 2 (32.2%). There were no significant between-cluster differences in age or height.

**Table 2:** Baseline characteristics of participants by cluster (n = 1,404).

Variable	Cluster 1 (Stable)	Cluster 2 (Adiposity-Increasing)	Cluster 3 (Adiposity-Decreasing)	χ2 or F	p-value (Post-hoc)	Total
Sex (Male/Female)	214/460	140/295	67/228	9.375	.009^*^	421/983
Age	45.00 ± 6.52	44.67 ± 6.90	44.27 ± 6.65	1.258	.284	44.74 ± 6.67
Height	162.33 ± 8.27	162.87 ± 8.30	161.85 ± 7.33	1.459	.233	162.40 ± 8.09
Weight	63.84 ± 11.87	64.20 ± 11.87	65.97 ± 13.34	3.217	.040^*^ (3 > 1)	64.39 ± 12.21
BMI	24.11 ± 3.30	24.09 ± 3.30	25.07 ± 4.01	9.060	<.001^**^ (3 > 1, 2)	24.30 ± 3.48
WC	82.12 ± 8.63	80.91 ± 9.24	85.63 ± 9.79	24.853	<.001^**^ (3 > 1, 2)	82.47 ± 9.23
HC	95.36 ± 5.64	94.99 ± 5.93	97.80 ± 6.44	22.871	<.001^**^ (3 > 1, 2)	95.76 ± 6.00
WHR	0.86 ± 0.06	0.85 ± 0.06	0.87 ± 0.06	14.007	<.001^**^ (3 > 1 > 2)	0.86 ± 0.06
WHtR	0.51 ± 0.05	0.50 ± 0.05	0.53 ± 0.06	34.825	<.001^**^ (3 > 1 > 2)	0.51 ± 0.05
SMM	24.19 ± 5.81	24.45 ± 5.86	24.08 ± 5.36	.423	.655	24.25 ± 5.73
BFM	19.78 ± 5.83	19.69 ± 6.23	22.02 ± 7.57	14.942	<.001^**^ (3 > 1, 2)	20.22 ± 6.42
PSQI	4.50 ± 2.75	4.93 ± 2.81	4.64 ± 3.12	3.067	.047^*^ (2 > 1)	4.67 ± 2.86
GPAQ	−0.03 ± 0.99	0.07 ± 1.07	−0.04 ± 0.90	1.671	.188	0.00 ± 1.00

Weight, BMI, WC, HC, WHR, and WHtR were used as input variables in cluster analysis.

Group comparisons were evaluated using Chi-square tests for categorical variables and ANOVA for continuous variables.

Post-hoc tests used Tukey’s HSD, or Games–Howell when Levene’s test indicated unequal variances.

GPAQ was standardized as a Z-score.

BMI: body mass index, WC: waist circumference, HC: hip circumference, WHR: waist-to-hip ratio, WHtR: waist-to-height ratio, SMM: skeletal muscle mass, BFM: body fat mass, PSQI: Pittsburgh Sleep Quality Index, GPAQ: Global Physical Activity Questionnaire

* *p* < .05; ** *p* < .001

Weight differed modestly between clusters (*p* = .040), whereas BMI, WC, HC, WHR, and WHtR differed more strongly (all *p* < .001). Cluster 3 had the highest mean values for these body-shape indicators.

Among body-composition indicators, BFM differed significantly between clusters (*p* < .001), with the highest mean BFM in Cluster 3; in contrast, SMM did not differ significantly between clusters (*p* = .655).

Among lifestyle indicators, PSQI differed significantly between clusters (*p* = .047), with the highest mean PSQI in Cluster 2. GPAQ scores did not differ significantly between clusters (*p* = .188).

These baseline body-shape, body-composition, and lifestyle variables, treated as external variables not used in the K-means algorithm, were used to characterize and interpret the three data-driven clusters.

Taken together, these patterns indicate that Cluster 3 comprised participants with the highest baseline adiposity who subsequently showed decreasing trajectories, whereas Clusters 1 and 2 started from lower and broadly similar adiposity levels but diverged into stable versus adiposity-increasing trajectories over the 2-year follow-up.

### Comparison of changes in body composition and lifestyle habits

When changes from V1 to V2 were compared across clusters, there were significant between-cluster differences in most variables (Table 3).

**Table 3:** One-way ANOVA for changes in body shape and lifestyle variables by cluster.

Variable	Cluster 1 (Stable)	Cluster 2 (Adiposity-Increasing)	Cluster 3 (Adiposity-Decreasing)	F	p-value (Post-hoc)	Total
ΔBMI	0.04 ± 0.79	1.21 ± 1.05	−1.18 ± 1.33	505.634	<.001^**^ (2 > 1 > 3)	0.15 ± 1.32
ΔWHR	0.01 ± 0.03	0.04 ± 0.03	−0.03 ± 0.04	384.532	<.001^**^ (2 > 1 > 3)	0.01 ± 0.04
ΔWHtR	0.01 ± 0.01	0.04 ± 0.02	−0.03 ± 0.02	1575.381	<.001^**^ (2 > 1 > 3)	0.01 ± 0.03
ΔWC	1.37 ± 2.37	7.23 ± 3.23	−5.30 ± 3.53	1629.237	<.001^**^ (2 > 1 > 3)	1.79 ± 5.33
ΔHC	0.73 ± 2.00	3.92 ± 2.36	−2.69 ± 2.64	762.352	<.001^**^ (2 > 1 > 3)	1.00 ± 3.26
ΔWeight	0.29 ± 1.99	3.43 ± 2.74	−3.02 ± 3.49	546.266	<.001^**^ (2 > 1 > 3)	0.57 ± 3.48
ΔSMM	0.02 ± 0.79	0.31 ± 0.91	−0.39 ± 0.88	59.083	<.001^**^ (2 > 1 > 3)	0.02 ± 0.88
ΔBFM	0.28 ± 1.97	2.95 ± 2.48	−2.39 ± 3.23	429.082	<.001^**^ (2 > 1 > 3)	0.55 ± 3.10
ΔPSQI	0.77 ± 3.32	0.66 ± 3.45	1.28 ± 3.75	3.057	.047^*^ (NS)	0.84 ± 3.46
ΔGPAQ	−0.02 ± 0.93	−0.13 ± 1.01	0.22 ± 1.11	11.078	<.001^**^ (3 > 1, 2)	0.00 ± 1.00

Weight, BMI, WC, HC, WHR, and WHtR were used as input variables in cluster analysis.

Group comparisons were evaluated using Chi-square tests for categorical variables and ANOVA for continuous variables.

Post-hoc tests used Tukey’s HSD, or Games–Howell when Levene’s test indicated unequal variances.

Δ denotes change between V2 and V1.

GPAQ was standardized as a Z-score.

BMI: body mass index, WC: waist circumference, HC: hip circumference, WHR: waist-to-hip ratio, WHtR: waist-to-height ratio, SMM: skeletal muscle mass, BFM: body fat mass, PSQI: Pittsburgh Sleep Quality Index, GPAQ: Global Physical Activity Questionnaire

* *p* < .05; ** *p* < .001

Among body-shape indicators, Δweight, ΔBMI, ΔWC, ΔHC, ΔWHR, and ΔWHtR all differed significantly between clusters (all *p* < .001). The largest increases were observed in Cluster 2 (Adiposity-Increasing), whereas clear decreases were observed in Cluster 3 (Adiposity-Decreasing). Cluster 1 (Stable) showed minimal overall change.

Among body-composition indicators, ΔSMM differed significantly between clusters, increasing in Cluster 2 and decreasing in Cluster 3 (*p* < .001). ΔBFM also differed significantly between clusters, increasing in Cluster 2 and decreasing in Cluster 3 (*p* < .001).

Among lifestyle indicators, ΔPSQI differed significantly between clusters (*p* = .047), although post-hoc comparisons were not significant. ΔGPAQ increased significantly in Cluster 3 compared with Clusters 1 and 2 (*p* < .001).

### Baseline indicator analysis for cluster prediction

To examine baseline (V1) indicators associated with cluster membership, multinomial logistic regression was performed with Cluster 3 as the reference group. Analyses were conducted using a crude model and an age- and sex-adjusted model  (Table 4).

When Cluster 1 was compared with Cluster 3, BFM was a significant predictor. Lower BFM was associated with higher odds of Cluster 1 membership (crude OR = 0.95, 95% CI 0.93–0.97, *p* < .001; adjusted OR = 0.96, 95% CI 0.94–0.99, *p* < .05).

Similarly, when Cluster 2 was compared with Cluster 3, BFM remained significant; lower BFM was associated with higher odds of Cluster 2 membership (crude OR = 0.94, 95% CI 0.92–0.97, *p* < .001; adjusted OR = 0.95, 95% CI 0.93–0.98, *p* < .001).

In contrast, SMM, PSQI, and GPAQ were not significant predictors in any between-cluster comparisons (all *p* > .05).

**Table 4:** Multinomial logistic regression of change indicators for predicting cluster membership.

Variable	Crude		Adjusted	
	OR (95% CI)	p-value	OR (95% CI)	p-value
Cluster 1 (Stable)				
ΔBFM	1.79 (1.64, 1.96)	<.001^**^	1.82 (1.66, 1.99)	<.001^**^
ΔSMM	2.60 (2.08, 3.26)	<.001^**^	2.69 (2.13, 3.39)	<.001^**^
ΔPSQI (Ref. = MCID < 2.5)				
ΔPSQI MCID ≥ 2.5	1.38 (0.95, 1.98)	.087	1.43 (0.99, 2.08)	.056
ΔGPAQ (Ref. = Q4, highest quartile of change in GPAQ)				
ΔGPAQ Q1	1.40 (0.88, 2.23)	.155	1.41 (0.88, 2.26)	.152
ΔGPAQ Q2	1.39 (0.87, 2.21)	.168	1.41 (0.88, 2.25)	.152
ΔGPAQ Q3	1.19 (0.76, 1.86)	.460	1.24 (0.78, 1.95)	.360
Cluster 2 (Adiposity-Increasing)				
ΔBFM	3.79 (3.32, 4.33)	<.001^**^	3.87 (3.39, 4.43)	<.001^**^
ΔSMM	6.62 (4.94, 8.88)	<.001^**^	7.01 (5.20, 9.47)	<.001^**^
ΔPSQI (Ref. = MCID < 2.5)				
ΔPSQI MCID ≥ 2.5	2.09 (1.29, 3.38)	.003^*^	2.21 (1.36, 3.60)	.001^*^
ΔGPAQ (Ref. = Q4, highest quartile of change in GPAQ)				
ΔGPAQ Q1	2.14 (1.17, 3.89)	.013^*^	2.15 (1.17, 3.94)	.013^*^
ΔGPAQ Q2	1.66 (0.91, 3.04)	.097	1.70 (0.93, 3.12)	.086
ΔGPAQ Q3	1.33 (0.74, 2.41)	.342	1.38 (0.76, 2.51)	.288

All analyses were conducted with Cluster 3 (Adiposity-Decreasing) as the reference group.

Crude: unadjusted; Adjusted: adjusted for sex and age.

Δ denotes change between V2 and V1.

ΔPSQI was dichotomized at the minimal clinically important difference (MCID) of 2.5 (≥ 2.5 = 2, < 2.5 = 1; reference category = 1).

ΔGPAQ (Z-score) was categorized into quartiles (Q1–Q4), with Q4 (highest quartile) as the reference category.

OR: odds ratio; CI: confidence interval; VIF: variance inflation factor; BFM: body fat mass; SMM: skeletal muscle mass; PSQI: Pittsburgh Sleep Quality Index; GPAQ: Global Physical Activity Questionnaire; MCID: minimal clinically important difference.

* *p* < .05; ** *p* < .001.

Model fit testing indicated statistical significance (model χ2, p < .001). The Nagelkerke pseudo-R2 was.581, which was substantially higher than that of the baseline-indicator models (0.041), indicating greater explanatory power for cluster membership when using change indicators (Table 4, R2 = .041).

### Analysis of changes for cluster prediction

To assess change indicators associated with cluster membership, multinomial logistic regression was again performed with Cluster 3 as the reference group using crude and age- and sex-adjusted models (Table 5).

When Cluster 1 was compared with Cluster 3, ΔBFM and ΔSMM were significant predictors: increases in either measure were associated with higher odds of Cluster 1 membership (ΔBFM: crude OR = 1.79, 95% CI 1.64–1.96, *p* < .001; adjusted OR = 1.82, 95% CI 1.66–1.99, *p* < .001; ΔSMM: crude OR = 2.60, 95% CI 2.08–3.26, *p* < .001; adjusted OR = 2.69, 95% CI 2.13–3.39, *p* < .001). ΔPSQI and ΔGPAQ were not significant for Cluster 1 (all *p* > .05).

Similarly, when Cluster 2 was compared with Cluster 3, ΔBFM and ΔSMM were significant: increases were associated with higher odds of Cluster 2 membership (ΔBFM: crude OR = 3.79, 95% CI 3.32–4.33, *p* < .001; adjusted OR = 3.87, 95% CI 3.39–4.43, *p* < .001; ΔSMM: crude OR = 6.62, 95% CI 4.94–8.88, *p* < .001; adjusted OR = 7.01, 95% CI 5.20–9.47, *p* < .001). ΔPSQI was significant for Cluster 2 only: a clinically meaningful worsening of sleep quality (ΔPSQI ≥ 2.5) was associated with higher odds of Cluster 2 membership (crude OR = 2.09, 95% CI 1.29–3.38, *p* < .05; Adjusted OR = 2.21, 95% CI 1.36–3.60, *p* < .05). ΔGPAQ reached significance only for Cluster 2, where the lowest quartile (Q1) was associated with higher odds of Cluster 2 membership (crude OR = 2.14, 95% CI 1.17–3.89, *p* < .05; Adjusted OR = 2.15, 95% CI: 1.17–3.94, *p* < .05). In the sex-stratified analyses, changes in body fat mass and skeletal muscle mass were consistently associated with cluster membership in both men and women. Associations involving changes in sleep quality and physical activity were less consistent across sex strata (S3 Table).

**Table 5:** Multinomial logistic regression of baseline indicators for predicting cluster membership.

Variable	Crude		Adjusted	
	OR (95% CI)	p-value	OR (95% CI)	p-value
Cluster 1 (Stable)				
BFM	.95(0.93, 0.97)	<.001^**^	.96(0.94, 0.99)	.002^*^
SMM	1.02(0.99, 1.04)	.213	.95(0.90, 1.00)	.053
PSQI	1.09(0.82, 1.45)	.556	1.09(0.82, 1.46)	.540
Z GPAQ	1.04(0.89, 1.21)	.648	1.04(0.89, 1.21)	.629
Cluster 2 (Adiposity-Increasing)				
BFM	.94(0.92, 0.97)	<.001^**^	.95(0.93, 0.98)	<.001^**^
SMM	1.03(1.00, 1.06)	.060	.98(0.92, 1.04)	.455
PSQI	.91(0.67, 1.24)	.560	.92(0.67, 1.25)	.579
GPAQ	1.12(0.96, 1.31)	.147	1.13(0.96, 1.32)	.140

All analyses were conducted with Cluster 3 (Adiposity-Decreasing) as the reference group.

Crude: unadjusted, Adjusted: adjusted for sex and age

GPAQ was standardized as a Z-score.

PSQI was dichotomized at a cutoff of 5 points (≥ 5 = 1; poor sleep, < 5 = 2; good sleep, reference category = 2).

OR: odds ratio, CI: confidence interval, VIF: variance inflation factor, BFM: body fat mass, SMM: skeletal muscle mass, PSQI: Pittsburgh Sleep Quality Index, GPAQ: Global Physical Activity Questionnaire

* *p* < .05; ** *p* < .001

The baseline-indicator models had a low Nagelkerke pseudo-R2 (0.041), indicating limited explanatory power for cluster membership, although the overall models were statistically significant (model χ2, p < .001). Accordingly, these baseline models should be interpreted as having limited ability to distinguish cluster membership.

## Discussion

In this study, 2-year follow-up data from adult men and women in South Korea were analyzed using cluster analysis based on changes in body shape. Using K-means clustering, three clusters were identified—Stable, Adiposity-Increasing, and Adiposity-Decreasing—with distinct baseline characteristics and trajectories. Among body-composition indicators, ΔBFM and ΔSMM showed the clearest associations with cluster membership. Among lifestyle indicators, ΔPSQI and ΔGPAQ were significantly associated with membership in the Adiposity-Increasing group. These findings suggest that changes in body shape and lifestyle habits are associated with obesity-related trajectories. Consistent with this pattern, the multinomial models that included only baseline indicators showed a very low Nagelkerke pseudo-R^2^ (0.041), whereas models incorporating change indicators yielded a substantially higher pseudo-R^2^ (0.581). This contrast suggests that cross-sectional baseline measures had limited ability to distinguish cluster membership, whereas longitudinal changes in body composition and lifestyle were more strongly associated with the derived trajectories. Accordingly, these multinomial models should be viewed as explanatory tools for understanding group-level patterns rather than as high-performance prediction models for individual risk.

In the cluster-specific pattern analysis, the Adiposity-Decreasing group had the highest baseline body-shape and body-composition indicators but showed a decreasing pattern during follow-up. The Adiposity-Increasing group differed little from the Stable group at baseline but showed overall increases in adiposity-related indicators during follow-up. Likewise, prior studies have reported that weight gain is more prominent among healthy-weight or mildly obese groups, whereas severely obese groups tend to decrease or stabilize [35]. In other words, less-obese groups may be more likely to progress toward greater adiposity, which is a clinically important observation.

Over the 2-year follow-up, the Adiposity-Increasing group showed the highest values for ΔSMM and ΔBFM, whereas the Adiposity-Decreasing group showed the lowest values, mirroring changes in body-shape indicators. Hootman et al. (2017) reported that weight gain is accompanied by increased fat mass [36], and Heymsfield et al. (2014) demonstrated that weight loss can involve reductions in both fat and lean mass [37]. Together, these findings suggest that body-composition indicators are closely related to body-shape indicators. The present results support that changes in body shape are closely linked to changes in fat and muscle mass. In particular, BFM (baseline and change) was significant in all models, suggesting broad associations with body-shape change. Notably, the strength of association was weaker for baseline BFM (OR, 0.95–0.96), supporting a greater emphasis on longitudinal change than on absolute baseline values.

ΔSMM showed an especially strong association with cluster membership. Relative to the Adiposity-Decreasing group, increases in ΔSMM were significantly associated with membership in both the Stable and Adiposity-Increasing groups, and ΔSMM had the largest ORs (Stable: 2.60–2.69; Adiposity-Increasing: 6.62–7.01) among all indicators. These odds ratios should be interpreted in light of the measurement scale, because the models used a 1-kg change in SMM as the unit; more modest changes in muscle mass would therefore correspond to proportionally smaller changes in the odds of cluster membership. Changes in muscle mass are associated with aging and decreased activity levels [38]. Given the mean participant age of 44.7 years, age-related changes in muscle mass may have contributed to the observed associations with cluster membership [39]. In the present study, increases in SMM accompanied the Adiposity-Increasing trajectory, indicating that muscle mass should be considered together with fat mass when interpreting weight and body-shape changes.

Among lifestyle indicators, ΔPSQI and ΔGPAQ were significantly associated with membership in the Adiposity-Increasing trajectory group. A ΔPSQI meeting the MCID threshold (≥2.5) was more strongly associated with the Adiposity-Increasing group. Previous studies have reported that impaired sleep quality is associated with weight gain and with changes in appetite-regulating hormones such as leptin and ghrelin [40,41]. In the present study, the association between ΔPSQI and body-shape trajectories is consistent with the hypothesis that changes in sleep quality may be linked to physiological pathways influencing body-shape change; however, given the observational design, these potential mechanisms cannot be confirmed. Thus, clinically meaningful deterioration in sleep quality was associated with a higher likelihood of belonging to the Adiposity-Increasing trajectory group, and this relationship should be interpreted as an association rather than a causal effect.

ΔGPAQ was significant only for the lowest quartile (Q1; decrease of ≥1,200 metabolic equivalent of task [MET]-minutes per week). Under WHO criteria, this represents a decrease in activity level of at least one category [42]. In other words, large decreases in activity level were significantly associated with membership in the Adiposity-Increasing group.

In summary, the Adiposity-Increasing group was characterized by overall increases in body-shape indicators, worsening sleep quality (ΔPSQI), and decreasing activity levels (ΔGPAQ). In contrast, the Adiposity-Decreasing group exhibited decreases in body-shape indicators during follow-up but showed the highest adiposity-related indicators at baseline, and ΔSMM was strongly associated with membership in this cluster. These findings suggest that, in addition to weight change, maintenance of body composition and changes in lifestyle factors are associated with changes in adiposity-related indicators over time.

From a public health perspective, tracking sleep quality and changes in physical activity alongside body-shape changes may be useful for early identification of at-risk groups and for designing customized interventions. For example, similar degrees of weight gain may have different health implications depending on whether they are accompanied by regular exercise versus sedentary behavior and worsening sleep quality. In this context, one strength of this study is that patterns of body-shape changes were analyzed longitudinally using 2-year follow-up data from male and female Korean adults. Unlike many previous studies limited to cross-sectional designs, this study examined dynamic relationships between body shape and lifestyle factors by incorporating changes over time. Moreover, by analyzing not only conventional body-shape indicators (BMI, WC, HC, WHR, and WHtR) but also body-composition indicators (BFM and SMM) and lifestyle indicators (PSQI and GPAQ), this study used a multidimensional approach. This integrative analysis provides a more detailed understanding of how co-occurring changes in body shape, body composition, and lifestyle factors relate to obesity-related trajectories.

Beyond the Korean context, our findings align with a growing body of work using cluster- or trajectory-based approaches to characterize long-term patterns of adiposity and their correlates in diverse populations. For example, in the Brazilian Longitudinal Study of Adult Health (ELSA-Brasil), Pimenta et al. (2023) identified distinct body-shape trajectories from childhood to mid-adulthood and reported that these patterns were associated with birth weight, current BMI, and sociodemographic conditions rather than representing fixed clinical categories [43]. Similarly, analyses from the China Health and Nutrition Survey by Dang et al. (2023) demonstrated that heterogeneity in BMI trajectories across adulthood can be partially explained by differences in life-course socioeconomic position and related behavioral factors [44]. Taken together, these studies and our results in Korean adults suggest that cluster-based characterization of body-shape change and its behavioral and compositional correlates may be informative across settings. Nevertheless, the specific trajectory patterns and their determinants are likely to vary by culture and environment, and replication in other Asian and non-Asian populations will be required before these findings can be generalized beyond the present cohort.

### Limitations

This study has a few limitations.

First, the 2-year follow-up period was relatively short, which limited our ability to evaluate long-term body-shape changes and downstream health outcomes, and part of this period overlapped with the COVID-19 pandemic in Korea; therefore, some observed changes may partly reflect temporary pandemic-related influences rather than typical long-term trajectories.

Second, analyses were based on a complete-case dataset of 1,404 participants out of approximately 2,000 individuals enrolled at baseline. Baseline comparisons indicated that included and excluded individuals were similar in major anthropometric and body-composition measures, with only small differences in age and hip circumference; nonetheless, selection bias cannot be entirely ruled out.

Third, participants were middle-aged adults undergoing health examinations, and women were overrepresented (approximately 70% of the sample), which limits generalizability to the wider population and to male-dominant groups. More diverse samples with respect to sex, age, socioeconomic status, and underlying disease are needed.

Fourth, sleep and physical activity were assessed using self-report questionnaires, so recall bias are possible. Objective measurements from wearable devices or digital health data would strengthen future research.

Fifth, no dedicated a priori power calculation was performed for the specific clustering and multinomial logistic regression analyses; the sample size was determined by the KDCC cohort design. In addition, dichotomization or categorization of some predictors may have reduced statistical power, so effect estimates for lifestyle variables should be interpreted with caution.

Sixth, the associations of ΔBFM and ΔSMM with cluster membership should be interpreted with caution because ΔWeight was included among the clustering variables. Since body weight reflects fat mass, lean mass, and other components, the large odds ratios for ΔBFM and ΔSMM and the increase in Nagelkerke pseudo-R^2^ from 0.041 to 0.581 may partly reflect overlap with the clustering variables rather than independent prediction of cluster membership.

Seventh, the clusters identified by K-means represent data-driven partitions of body-shape change rather than biological or clinical subtypes. The primary algorithm was applied to unstandardized change scores for anthropometric indicators on different scales, and this should be considered a methodological limitation. Although the sensitivity analysis using Z-score standardized variables showed high agreement with the primary cluster solution, alternative preprocessing choices, clustering methods, or values of k could yield different solutions. Thus, the three clusters should be interpreted as one plausible summary of body-shape trajectories in this cohort.

Finally, body composition was assessed using bioelectrical impedance analysis (InBody 770), which is sensitive to hydration status and does not directly quantify visceral fat or regional fat distribution. Direct measures of muscle function were also unavailable. As a result, we could not fully capture qualitative aspects of weight change, such as shifts in fat distribution or changes in muscle quality, beyond quantitative changes in total fat and muscle mass.

## Conclusions

In this study, 2-year follow-up data from Korean adults undergoing health examinations were used to derive clusters based on patterns of body-shape change and to evaluate associations of baseline indicators and their changes with cluster membership. Three clusters were identified (Stable, Adiposity-Increasing, and Adiposity-Decreasing). Changes in BFM and SMM were strongly associated with cluster membership. Worsening sleep quality and decreased physical activity were also associated with membership in the Adiposity-Increasing group.

These findings indicate that, rather than relying only on absolute values of body-shape indicators, integrating lifestyle factors and longitudinal patterns of change may be informative for assessing obesity-related trajectories. From clinical and public health perspectives, it may be useful to integrate dynamic changes in body shape and body composition with relevant lifestyle factors. Such integrative approaches could support a more detailed characterization of adiposity-related change patterns and inform tailored lifestyle interventions.

## Supporting information

S1 Table Cluster classification based on standardized changes in body shape parameters. This table presents the cluster centroids from the K-means clustering analysis repeated using Z-score standardized body-shape change indicators.
(DOCX)

S2 Table Agreement between primary and standardized-variable K-means cluster solutions. This table presents the cross-tabulation between the primary unstandardized K-means cluster solution and the standardized-variable K-means solution. Agreement was calculated as the proportion of participants classified into the same cluster category in both solutions.
(DOCX)

S3 Table Sex-stratified multinomial logistic regression analyses of change indicators associated with body-shape change clusters. This table presents sex-stratified multinomial logistic regression analyses using the same cluster definitions as in the main analysis, with adjustment for age.
(DOCX)

## Abbreviations

BMI: body mass index
WC: waist circumference
WHR: waist-to-hip ratio
WHtR: waist-to-height ratio
KDCC: Korean Medicine Daejeon Citizen Cohort
COVID-19: coronavirus disease 2019
HC: hip circumference
BFM: body fat mass
SMM: skeletal muscle mass
PSQI: Pittsburgh Sleep Quality Index
GPAQ: Global Physical Activity Questionnaire
OR: odds ratio
CI: confidence interval
VIF: variance inflation factor
MCID: minimal clinically important difference
