# Supplementary material for: Lifestyle factors and clustered patterns of body shape change in Korean adults: A longitudinal observational study
Source: PLoS One. 2026 Oct 15;21(10):e0360853. doi: 10.1371/journal.pone.0360853 (PMC13671638; doi:10.1371/journal.pone.0360853)
Supplement: Supplementary file 1 [file pone.0360853.s001.docx]

S1 Table. Cluster Classification Based on Standardized Changes in Body Shape Parameters (n=1,404)

| Variables | Cluster 1  (Stable) | Cluster 2  (Adiposity-Increasing) | Cluster 3  (Adiposity-Decreasing) |
| --- | --- | --- | --- |
| Total n (%) | 684 (48.7%) | 443 (31.6%) | 277 (19.7%) |
| Cluster centroids |  |  |  |
| Z-ΔBMI | -0.08 | 0.81 | -1.10 |
| Z-ΔWHR | -0.11 | 0.80 | -1.01 |
| Z-ΔWHtR | -0.12 | 1.03 | -1.36 |
| Z-ΔWC | -0.12 | 1.03 | -1.36 |
| Z-ΔHC | -0.06 | 0.79 | -1.11 |
| Z-ΔWeight | -0.08 | 0.81 | -1.10 |
| Cluster centroids of body shape change variables | | | |
