# Supplementary material for: Lifestyle factors and clustered patterns of body shape change in Korean adults: A longitudinal observational study
Source: PLoS One. 2026 Oct 15;21(10):e0360853. doi: 10.1371/journal.pone.0360853 (PMC13671638; doi:10.1371/journal.pone.0360853)
Supplement: Supplementary file 2 [file pone.0360853.s002.docx]

**S2 Table.** Agreement between primary and standardized-variable K-means cluster

| Primary cluster | Standardized  Stable | Standardized  Adiposity-Increasing | Standardized  Adiposity-Decreasing | Agreement |
| --- | --- | --- | --- | --- |
| Stable | 630 (93.5%) | 35 (5.2%) | 9 (1.3%) | 630/674 (93.5%) |
| Adiposity-Increasing | 27 (6.2%) | 408 (93.8%) | 0 (0.0%) | 408/435 (93.8%) |
| Adiposity-Decreasing | 27 (9.2%) | 0 (0.0%) | 268 (90.8%) | 268/295 (90.8%) |
| Overall | 684 (48.7%) | 443 (31.6%) | 277 (19.7%) | 1306/1404 (93.0%) |
| Values are n (% within each primary cluster).  Agreement was calculated as the proportion of participants classified into the same cluster category in both the primary and standardized-variable K-means solutions. | | | | |
