# Supplementary material for: Lifestyle factors and clustered patterns of body shape change in Korean adults: A longitudinal observational study
Source: PLoS One. 2026 Oct 15;21(10):e0360853. doi: 10.1371/journal.pone.0360853 (PMC13671638; doi:10.1371/journal.pone.0360853)
Supplement: Supplementary file 3 [file pone.0360853.s003.docx]

S3 Table. Sex-stratified multinomial logistic regression analyses of change indicators associated with body-shape change clusters

| Variable | Male | | Female | |
| --- | --- | --- | --- | --- |
|  | Adjusted | | | |
|  | OR (95% CI) | p-value | OR (95% CI) | p-value |
| Cluster 1 (Stable) | | | | |
| ΔBFM | 2.51 (1.96, 3.22) | < .001^**^ | 1.68 (1.52, 1.86) | < .001^**^ |
| ΔSMM | 3.23 (2.00, 5.20) | < .001^**^ | 2.70 (2.04, 3.58) | < .001^**^ |
| ΔPSQI (Ref. = MCID < 2.5) | | | | |
| ΔPSQI MCID ≥ 2.5 | 2.47 (1.09, 5.60) | .030^*^ | 1.22 (0.80, 1.87) | .355 |
| ΔGPAQ (Ref. = Q4, highest quartile of change in GPAQ) | | | | |
| ΔGPAQ Q1 | 0.73 (0.24, 2.19) | .575 | 1.67 (0.98, 2.82) | .058 |
| ΔGPAQ Q2 | 0.50 (0.17, 1.42) | .192 | 1.83 (1.07, 3.13) | .027^*^ |
| ΔGPAQ Q3 | 0.46 (0.16, 1.32) | .150 | 1.62 (0.97, 2.70) | .066 |
| Cluster 2 (Adiposity-Increasing) | | | | |
| ΔBFM | 5.91 (4.29, 8.14) | < .001^**^ | 3.50 (3.01, 4.07) | < .001^**^ |
| ΔSMM | 7.38 (4.16, 13.06) | < .001^**^ | 8.37 (5.72, 12.23) | < .001^**^ |
| ΔPSQI (Ref. = MCID < 2.5) | | | | |
| ΔPSQI MCID ≥ 2.5 | 3.09 (1.11, 8.56) | .030^*^ | 2.10 (1.19, 3.71) | .010^*^ |
| ΔGPAQ (Ref. = Q4, highest quartile of change in GPAQ) | | | | |
| ΔGPAQ Q1 | 2.17 (0.58, 8.05) | .249 | 1.68 (0.83, 3.39) | .149 |
| ΔGPAQ Q2 | 0.71 (0.20, 2.59) | .604 | 1.98 (0.98, 4.00) | .056 |
| ΔGPAQ Q3 | 0.64 (0.16, 2.48) | .516 | 1.53 (0.78, 2.99) | .221 |
| All analyses were conducted with Cluster 3 (Adiposity-Decreasing) as the reference group. Adjusted: adjusted for age. Δ denotes change between V2 and V1. ΔPSQI was dichotomized at the minimal clinically important difference (MCID) of 2.5 (≥ 2.5 = 2, < 2.5 = 1; reference category = 1). ΔGPAQ (Z-score) was categorized into quartiles (Q1–Q4), with Q4 (highest quartile) as the reference category. OR: odds ratio; CI: confidence interval; VIF: variance inflation factor; BFM: body fat mass; SMM: skeletal muscle mass; PSQI: Pittsburgh Sleep Quality Index; GPAQ: Global Physical Activity Questionnaire; MCID: minimal clinically important difference.  *p < .05; **p < .001 | | | | |
